# Using HIV Surveillance Laboratory Data to Identify Out-of-Care Patients

**DOI:** 10.1007/s10461-017-1742-5

**Published:** 2017-03-06

**Authors:** John Christian Hague, Betsey John, Linda Goldman, Kshema Nagavedu, Sophie Lewis, Rebecca Hawrusik, Serena Rajabiun, Noelle Cocoros, H. Dawn Fukuda, Kevin Cranston

**Affiliations:** 1 Massachusetts Department of Public Health, Boston, MA USA; 2 Boston University School of Public Health, Boston, MA USA

**Keywords:** HIV laboratory surveillance, Out-of-care, Care engagement, Linkage

## Abstract

HIV-associated laboratory tests reported to public health surveillance have been used as a proxy measure of care engagement of HIV+ individuals. As part of a Health Resources and Services Administration (HRSA) Special Projects of National Significance (SPNS) Initiative, the Massachusetts Department of Public Health (MDPH) worked with three pilot clinical facilities to identify HIV+ patients whose last HIV laboratory test occurred at the participating facility but who then appeared to be out of care, defined as an absence of HIV laboratory test results reported to MDPH for at least 6 months. The clinical facilities then reviewed medical records to determine whether these patients were actually not in care, or if there was another reason that they did not have a laboratory test performed, and provided feedback to MDPH on each of the presumed out-of-care patients. In the first year of the pilot project, 37% of patients who appeared to be out of care based on laboratory data were confirmed to be out of care after review of clinical health records. Of those patients who were confirmed to be out of care, 55% had a subsequent laboratory test within 3 months, and 72% had a laboratory test within 6 months, indicating that they had re-engaged with a care provider. MDPH found that it was essential to have clinical staff confirm the care status of patients who were presumed to be out of care based on surveillance data.

## Introduction

Engagement in HIV care and treatment has been shown to contribute to improved health outcomes and reduced risk of onward HIV transmission [1]. The U.S. Department of Health and Human Services recommends that HIV+ individuals receive viral load testing at least every 6 months [2]. HIV-associated laboratory tests that are reported to public health surveillance have been used as a proxy measure of care engagement of HIV+ individuals [3–5].

In 2012, the Massachusetts Department of Public Health (MDPH) received funding from the federal Health Resources and Services Administration (HRSA), under a Special Projects of National Significance (SPNS) grant to pilot a novel, surveillance-driven linkage and retention intervention. The intervention used electronic laboratory reports (ELR) received by the Massachusetts HIV/AIDS Surveillance Program (MHASP) within MDPH, to identify patients who appeared to be out of care (OOC) at three pilot healthcare facilities. MHASP epidemiologists notified designated staff at those facilities to ascertain the true care status of the individuals based on clinical information from the medical care team. Drawing on information from both MHASP and medical records, this intervention aimed to: (1) identify how accurately surveillance data alone could identify OOC individuals; and (2) communicate patients’ OOC status to healthcare providers who could then make efforts to re-engage them in care.

This article describes the processes implemented by MHASP and health care facilities, early results of the intervention including how accurately surveillance data identified OOC patients, and strategies developed to successfully identify patients who were OOC and effectively target re-engagement activities. MDPH will use the findings to improve outreach and re-engagement services for persons with established HIV disease, and those newly diagnosed with HIV infection.

## Methods

Per Massachusetts disease reporting regulations, MHASP receives all positive HIV antibody laboratory test results, as well as CD4+ T-lymphocyte counts and HIV viral load laboratory tests regardless of result value. The majority of laboratory results are received electronically within three days of the test result date, and paper laboratory results are received within two weeks and entered into a unified database upon receipt. Combined with information about patients’ current residence and vital status, MHASP can use laboratory data to identify HIV+ individuals receiving care in Massachusetts. Those without recent laboratory test results may be OOC.

The population under study included individuals whose last HIV laboratory test occurred at one of three participating healthcare facilities, which included two of the largest medical centers in Massachusetts and one federally-qualified community health center. These patients were considered to have last been in care at one of these facilities and were therefore eligible for inclusion in the study. HIV+ individuals who did not have a CD4+ and/or viral load test for more than 6 months were “presumed OOC”, using laboratory tests as a proxy for an HIV care visit. MHASP generated presumed OOC line lists based on these criteria on the last day of each month and sent the lists to key staff at each of the pilot facilities.

**Fig. 1:**
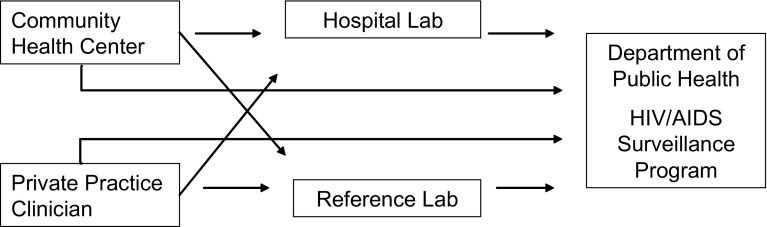
Flow diagram of laboratory test ordering from facilities in Massachusetts. Laboratory tests are sometimes sent through different facilities or reference laboratories. When the laboratory result is reported to MHASP, the “Facility Name” will sometimes reflect the facility where the sample was tested, not necessarily the ordering facility. However, the “Provider Name” field is a more accurate means of identifying the correct ordering facility

The “Facility Name” field on laboratory results often shows inaccurate or incomplete information due to the workflows associated with laboratory sample processing (Fig. 1). Therefore, to correctly identify which laboratory tests were ordered by the participating facilities, each facility provided a list of all clinicians who could order HIV laboratory tests and updated this list each month. A SAS program (SAS Institute, Cary, NC) was created to extract all laboratory tests associated with these ordering providers, accounting for spelling and name structure variations. We then used the “Provider Name” field in the laboratory report to correctly select those tests which were ordered by providers at the participating facilities.

The presumed OOC line lists were sent to each facility via encrypted, password-protected USB drives using an overnight courier service. Facility staff investigated the list of laboratory-record-generated presumed OOC patients by searching medical records and discussing patients’ care status with clinicians and case managers to determine whether they were confirmed OOC, or if there was clinical information indicating that they were not OOC. An encrypted line list was then sent back to MHASP describing the confirmed care status of each presumed OOC patient. The feedback about patients’ care status informed the next month’s line lists, such that patients determined to be in care would not appear on the subsequent line list. In this process, the facility records were considered the gold standard for the OOC determination.

Staff at each facility attempted to re-engage confirmed OOC patients following existing facility standard of care and linkage protocols. For presumed OOC patients who were not confirmed OOC, the facility staff reported one of the following potential reasons for the absence of a laboratory test report:

1. Patient had a clinic visit without laboratory testing
2. Patient had an upcoming appointment
3. Patient did not require a clinic visit every 6 months, as directed by clinician (e.g., long-term successful adherence to antiretroviral therapy)
4. Patient was not enrolled in care at the facility (e.g., patient transferred care, lived out of state, was incarcerated, was discharged from care)
5. Another reason not OOC

Each month, MHASP monitored the proportion of presumed OOC patients who were confirmed OOC and not confirmed OOC. Preliminary analysis included an examination of this proportion confirmed OOC by demographic and risk/exposure mode categories and tested differences for statistical significance at the 0.05 level using the Chi square test. MHASP also examined patient outcomes by determining whether confirmed OOC patients had a subsequent laboratory test at 3 and 6 months, respectively, after appearing on the presumed OOC line list.

## Results

**Fig. 2:**
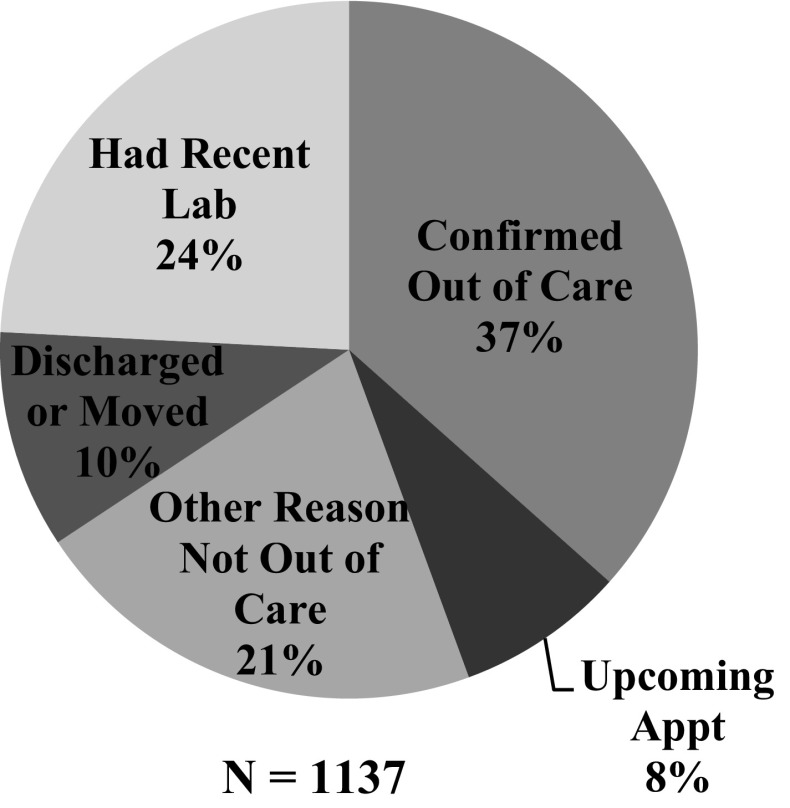
The proportion of patients confirmed OOC versus not confirmed OOC (with reason not OOC) after receiving clinical staff feedback regarding patients on the presumed OOC line list, June 2013–May 2014

During the first year of this pilot intervention, a total of 1137 individuals appeared on the presumed OOC line lists. Of these, 421 (37%) were confirmed OOC based on feedback from clinical staff (Fig. 2). Among those who were confirmed as not OOC, the most common reasons for appearing to be out of care were that the patient had a laboratory test that was received after the line list was generated or that was not reported to HIV surveillance (24%); or that the patient was directed by a clinician to wait >6 months between laboratory tests (for example, because the patient was on a stable regimen with established viral suppression) (21%). No statistically significant differences were noted between the proportion confirmed OOC versus proportion not confirmed by age, sex, race/ethnicity, and risk/exposure mode categories (by Chi square test, significance measured at p < 0.05) (Table 1). However, several qualitative differences are apparent: people of younger age, Hispanic ethnicity, and having a history of intravenous drug use were more likely confirmed OOC than not. Conversely, black (non-Hispanic) patients were more likely not confirmed OOC. Among patients who were confirmed OOC, 55% had a subsequent laboratory test within 3 months of appearing on the OOC line list, and 72% had a subsequent laboratory test within 6 months.

**Table 1:** Demographic and risk/exposure mode for patients who appeared on the presumed out-of-care line list

	Confirmed out-of-care	Not confirmed out-of-care
	N = 421	N = 716
Birth sex		
Male	261 (62)	451 (63)
Female	160 (38)	265 (37)
Age category		
20–29 years	21 (5)	29 (4)
30–39 years	76 (18)	93 (13)
40–49 years	122 (29)	179 (25)
50–59 years	151 (36)	279 (39)
60 and older	51 (12)	136 (19)
Race/ethnicity		
Non-Hispanic white	139 (33)	222 (31)
Non-Hispanic black	134 (32)	286 (40)
Hispanic/Latino	143 (34)	186 (26)
Other/unknown	5 (1)	22 (3)
Risk/exposure mode		
MSM	93 (22)	165 (23)
IDU	126 (30)	179 (25)
MSM/IDU	14 (3)	29 (4)
Heterosexual^a^	88 (21)	150 (21)
Presumed Heterosexual^b^	50 (12)	86 (12)
Other/unknown	50 (12)	107 (15)

*MSM* male sex with male, *IDU* injection drug user

^a^Heterosexual exposure includes high-risk heterosexual contact, defined as heterosexual contact with an MSM, IDU, or Person Living with HIV/AIDS

^b^Presumed heterosexual = females reported heterosexual contact, but not high-risk

## Discussion

Using state surveillance data as a proxy for identifying HIV+ patients who are OOC, we found that only 37% were confirmed OOC following clinical review. While we anticipated that many individuals identified as presumed OOC via surveillance data would not be truly out-of-care, the intervention appears to be a useful care monitoring tool for the participating clinical sites. The majority of confirmed OOC patients had a subsequent laboratory test, indicating that they had returned to care. Through ongoing discussions with the pilot sites, they reported that the line lists were a helpful tool in managing patients’ engagement in care.

MHASP identified three key elements of the line list process that were essential to successfully identifying confirmed OOC patients:

1. The use of “Provider Name” to determine the correct ordering facility for each laboratory result prior to generating the presumed OOC line list;
2. Establishment of a single point of contact at each participating clinical facility to receive the line lists and conduct follow-up; and,
3. Receipt of regular feedback from facility staff about which patients on the presumed OOC line lists were confirmed OOC.

### Use of Provider Name to Determine Correct Ordering Facility for Each Laboratory Result

The first key element in creating the OOC line lists was determining the correct ordering facility for each laboratory report so the line lists only contained patients who were last seen at each respective facility. Many healthcare facilities in Massachusetts process their laboratory tests through another facility or provider (Fig. 1). As a result, the ordering facility listed on the laboratory report may not be the same facility where the sample originated. When MHASP created OOC lists using the ordering *facility* on each laboratory report, only 10–30% of the patients on the line list were confirmed to be current patients at the facility (results varied by site). However, the name of ordering *provider* on these laboratory reports far more accurately reflected the corresponding facility, which facilitated accurate matching of patients to their care facility. When MHASP created OOC lists based on the ordering provider, 85–100% of the patients on the list were confirmed to be patients at the facility. Using up-to-date clinician rosters that were provided by each participating facility was essential for creating accurate OOC line lists.

### Establishment of a Single Point of Contact at Each Clinical Facility

The second key step of the line list procedure was having a single point of contact at each participating clinical facility who was responsible for following up on the presumed OOC line lists. These individuals were frequently data managers or nurses. In part, this project funded partial salary support with the expectation they would allocate a significant portion of their time to line list follow-up. This element of the intervention ensured that the presumed OOC line lists were processed in a timely and accurate manner, and that complete information was reported to MHASP.

### Receipt of Feedback from Facilities About Patients on the Presumed OOC Line Lists

The third key element of the line list procedure was receiving feedback from staff at clinical facilities about the care status of patients on the presumed OOC line lists. Although patients may appear to be OOC based on the frequency of their HIV-related laboratory tests, we learned that patients often have reasons for the apparent lapse in care. Furthermore, there is some time lag between receiving laboratory results, generating the line lists, and sending them to the facilities. During that time lag, some patients will have had a laboratory test indicating that they are not OOC. A smaller portion of the not confirmed OOC patients had a lab that was not sent to MHASP due to a quality issue with facility laboratory reporting, which prompted additional quality assurance follow up to address the issue.

Incorporating clinical information about patients reveals key information about their care patterns that cannot be ascertained through surveillance data alone.

We also learned one of the main reasons for patients being misclassified as OOC based on surveillance records was related to frequency of testing. Although national guidelines recommend viral load testing at least every 6 months, many providers participating in this pilot project reported patients with stable treatment and consistent undetectable viral load on whom viral load testing was done on a less frequent schedule.

OOC line lists proved to be an essential first step in identifying HIV+ patients who have fallen out of care. We observed strong evidence of re-engagement in care, with 72% of confirmed OOC patients returning to care within 6 months. In order for this intervention to be effective and sustainable, resources must be dedicated to create, process, and act upon the OOC line lists. On a large scale, this intervention would require considerable investment of resources. MDPH plans to build on these lessons to expand the use of OOC line lists to additional facilities in Massachusetts.
